# Taxifolin Resensitizes Multidrug Resistance Cancer Cells via Uncompetitive Inhibition of P-Glycoprotein Function

**DOI:** 10.3390/molecules23123055

**Published:** 2018-11-22

**Authors:** Hsiu-Ju Chen, Yun-Lung Chung, Chia-Ying Li, Ying-Tzu Chang, Charles C. N. Wang, Hsiang-Yen Lee, Hui-Yi Lin, Chin-Chuan Hung

**Affiliations:** 1 Department of Pharmacy, College of Pharmacy, China Medical University, 91 Hsueh-Shih Road, Taichung 40402, Taiwan; sharonchen5888@gmail.com (H.-J.C.); tel22336978@gmail.com (Y.-T.C.); 2 Research Assistant Center, Show Chwan Health Care System, 542, Sec 1, Chung-shan Rd., Changhua 500, Taiwan; p730912@hotmail.com; 3 Department of Medical Research and Development, Chang Bing Show Chwan Memorial Hospital, No.6, Lugong Rd., Lugang Town, Changhua 505, Taiwan; 4 School of Medicine, College of Medicine, Fu Jen Catholic University, No.510, Zhongzheng Rd., Xinzhuang Dist., New Taipei City 24205, Taiwan; B86401115@ntu.edu.tw; 5 Department of Surgery, Show Chwan Memorial Hospital, 542, Sec 1, Chung-shan Rd., Changhua 500, Taiwan; 6 Department of Surgery, Chang Bing Show Chwan Memorial Hospital, No.6, Lugong Rd. Lugang Town, Changhua 505, Taiwan; 7 Department of Bioinformatics and Medical Engineering, Asia University, 500, Lioufeng Rd., Wufeng, Taichung 41354, Taiwan; chaoneng.wang@gmail.com; 8 Department of Internal Medicine, Taipei Medical University Hospital, No. 252, Wuxing St, Xinyi District, Taipei City 110, Taiwan; plumlikesalt@gmail.com; 9 Department of Pharmacy, China Medical University Hospital, 2 Yude Road, Taichung 40447, Taiwan

**Keywords:** taxifolin, quercetin, P-glycoprotein, multidrug resistance, kinetic mechanism

## Abstract

P-glycoprotein (P-gp) effluxes lots of chemotherapeutic agents and leads to multidrug resistance (MDR) in cancer treatments. The development of P-gp inhibitors from natural products provide a potential strategy for the beneficial clinical outcomes. This study aimed to evaluate the effects of the natural flavonoid taxifolin, luteolin, (−)-gallocatechin, and (−)-catechin on human P-gp activity. The kinetic interactions and underlying mechanisms of taxifolin-mediated transporter inhibition were further investigated. The transporter inhibition ability was evaluated in human P-gp stable expression cells (*ABCB1*/Flp-In^TM^-293) by calcein-AM uptake assays. The kinetics study for P-gp inhibition was evaluated by doxorubicin and rhodamine123 efflux assays. The MDR reversal ability of taxifolin were performed by SRB assays to detect the cell viability in sensitive cancer cell line (HeLaS3), and resistant cancer cell line (KB-vin). Cell cycle analysis and *ABCB1* real-time RT-PCR were used for mechanical exploration. The results demonstrated that taxifolin decreased *ABCB1* expression in a concentration-dependent manner. The function of P-gp was inhibited by taxifolin through uncompetitive inhibition of rhodamine 123 and doxorubicin efflux. The combination of taxifolin significantly resensitized MDR cancer cells to chemotherapeutic agents. These results suggested that taxifolin may be considered as a potential P-gp modulator for synergistic treatment of MDR cancers.

## 1. Introduction

Multidrug resistance (MDR) is one of the main causes of cancer chemotherapy failure. The most recognized mechanism of resistance is the decreased accumulation of drugs due to increased efflux by ATP-binding cassette (ABC) efflux transporters [1]. Among the ABC transporters, P-glycoprotein (P-gp), encoded by *ABCB1* gene, is the main efflux transporter for a variety of chemotherapeutic agents. P-gp is a 170 kDa efflux membrane transporter which is widely distributed throughout the human body (intestines, placenta, kidney, liver and blood–brain barrier). In normal cells, P-gp is responsible for limiting the uptake of carcinogens, toxins, and other xenobiotics [2]. On the other hand, overexpression of P-gp in cancer cells has been associated with the MDR phenomenon [3]. Therefore, the development of P-gp inhibitors is considered as a promising strategy to overcome MDR cancer. Although a large number of P-gp inhibitors have been developed, the unexpected systemic toxicities and pharmacokinetic interactions raised serious concerns regarding clinical benefits [4]. Recently, the development of fourth generation P-gp inhibitors with safety advantages from natural products has gradually been valued.

Flavonoids, a vast group of natural products, are the secondary metabolites of polyphenols, and widely found in fruits, vegetables, seeds and tea. Previous data have shown that flavonoids display many pharmacological activities, including antioxidant, anti-inflammatory and especially anti-cancer properties [5]. In epidemiologic data and clinical trials, flavonoids exhibit beneficial effects on cancer prevention and treatment [6]. The inhibitory potency of flavonoids on P-gp efflux function has been investigated previously. Among flavonoids, quercetin and catechin have been proven to be P-gp modulators. Quercetin could inhibit P-gp expression to increase accumulation of chemotherapeutic agents in MDR cancer cell lines [7,8]. In addition, catechins inhibit the binding and the transport activity of P-gp [9]. Therefore, flavonoids are potential chemosensitizing agents to overcome MDR cancers.

Taxifolin and luteolin are flavonoids structurally similar to quercetin. Taxifolin was shown to have strong anti-oxidant activities and inhibit the synthesis of triglyceride, which protects cerebral ischemic reperfusion injury. In addition, taxifolin also exhibited antiproliferative effects and enhanced apoptosis of various cancer cells induced by anticancer agents [10,11,12]. Catechins, also called flavan-3-ols, are the major polyphenols found in green tea. Among these, (−)-epigallocatechin, (−)-epicatechin gallate, and (−)-epigallocatechin gallate have demonstrated their inhibitory effects on the P-gp function via increasing the accumulation of rhodamine 123 and they potentiate the cytotoxicity of vinblastine in the MDR cancer cells [9,13]. However, the ability of isomeric (−)-gallic catechins and (−)-catechins to inhibit P-gp function remains unknown.

The present study aims to investigate the effects of taxifolin, luteolin, (−)-gallocatechin, and (−)-catechin on P-gp transporter activity. After the primary screening, taxifolin exhibited the most effectiveness in P-gp efflux inhibition. Therefore, we performed in-depth studies of the kinetic interactions and elucidated the underlying mechanisms of taxifolin-mediated transporter inhibition. The MDR cancer reversal potency of taxifolin was further evaluated by combining with current chemotherapy drugs in MDR cancer cell lines.

## 2. Results

### 2.1. Primary Screen of Effects on P-gp Efflux Function

**Figure 1:**
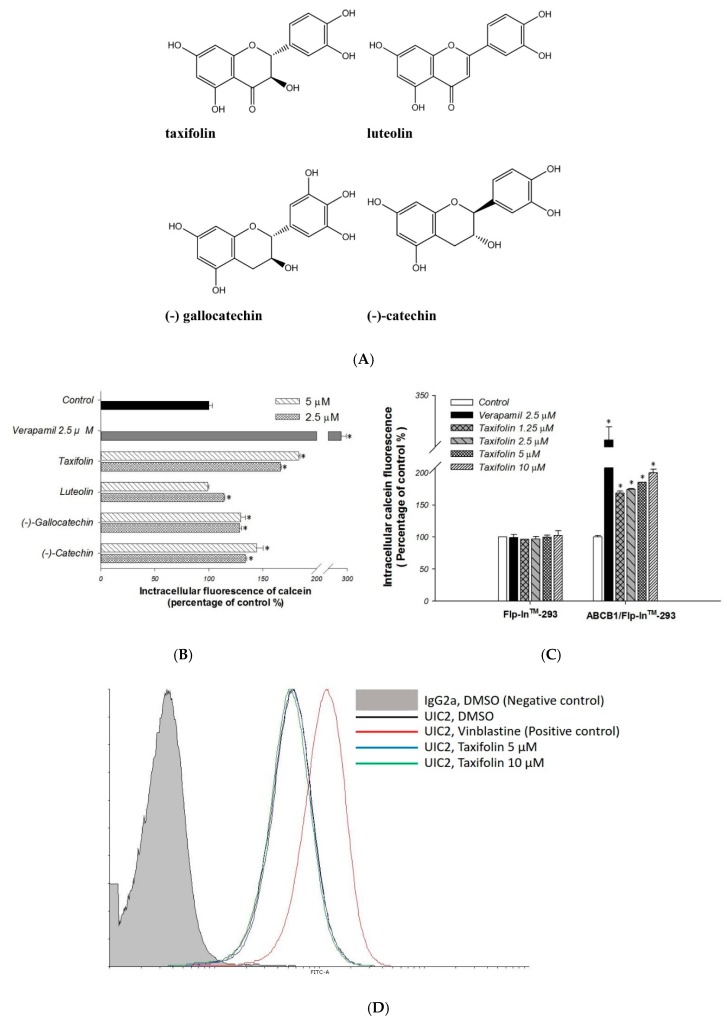
Evaluation of the effects of taxifolin, luteolin, (−)-gallocatechin, and (−)-catechin on P-gp transporter activity. (**A**) Chemical structures of taxifolin, luteolin, (−)-gallocatechin, and (−)-catechin. (**B**,**C**) The intracellular calcein fluorescence was significantly increased by taxifolin treatment in a dose dependent manner in *ABCB1*/Flp-In^TM^-293 cells. Verapamil 2.5 μM was used as a positive control. * denotes *p* < 0.05 as compared to untreated control. (**D**) In MDR1 shift assay, the UIC2 fluorescence intensity showed no difference between taxifolin treatment and solvent control as compare to the positive control vinblastine. Data presented as mean ± SE of at least three experiments, each in triplicate.

First, we performed the calcein-AM uptake assay for the primary screening of taxifolin, luteolin, (−)-gallocatechin, and (−)-catechin (Figure 1A) on P-gp efflux function. Calcein-AM is a non-fluoresent P-gp substrate and it would be converted to fluorescent calcein intracellularly. Therefore, the P-gp efflux function could be inversely correlated to intracellular calcein fluorescence. Verapamil was used as a standard P-gp inhibitor. The addition of taxifolin, luteolin, (−)-gallocatechin, or (−)-catechin, significantly increased the intracellular fluorescence as compared to the no-treatment control (Figure 1B). Among them, taxifolin exhibited the most favorable inhibitory effects on P-gp efflux function, and this effect was concentration-dependent (Figure 1C). Therefore, taxifolin was selected for further investigation.

### 2.2. The Molecular and Kinetic Mechanism of Interaction between Taxifolin and Human P-gp

To determine whether taxifolin is a P-gp substrate, MDR1 shift assay was performed. Treatment of taxifolin did not change the binding of P-gp conformation-sensitive antibody UIC2 as compare to the positive control vinblastine, indicating that taxifolin may not be a P-gp substrate (Figure 1D).

**Figure 2:**
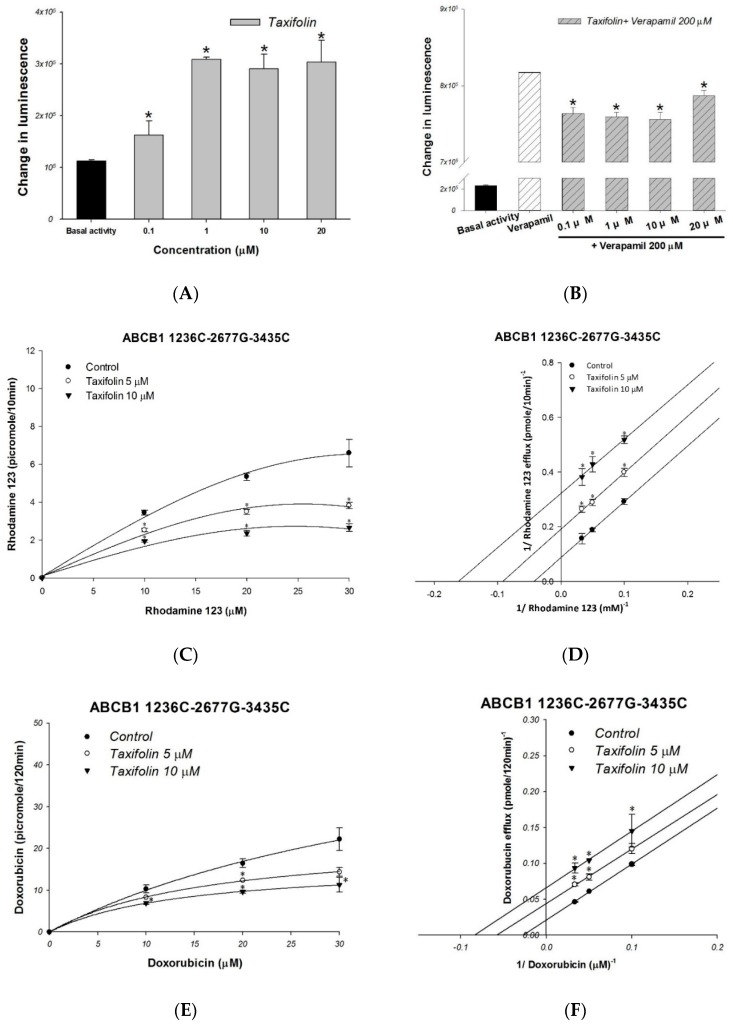
Analyses of the effect of taxifolin on P-gp ATPase activity and kinetic mechanisms of inhibition. P-gp ATPase activity was measured by Pgp-Glo^TM^ Assay System and data were analyzed in terms of RLUs. (**A**) Incubation with taxifolin (0.1–20 μM) increased the P-gp ATPase activity. (**B**) The verapamil-stimulated P-gp ATPase activity was decreased by taxifolin treatment. (**C**–**F**) P-gp inhibition kinetics analysis of taxifolin on rhodamine 123 efflux and doxorubicin efflux. The left panels show the dose-dependent effect of taxifolin on rhodamine 123 efflux (**C**) and doxorubicin efflux (**E**) followed the Michaelis–Menten kinetics, and the right panels demonstrate the Lineweaver−Burk plot analysis of rhodamine 123 efflux (**D**) and doxorubicin efflux (**F**). Data presented as mean ± SE of at least three experiments, each in triplicate. * denotes *p* < 0.05 as compared to untreated control in (**A**,**C**–**F**); In (**B**), * denotes *p* < 0.05 as compared to verapamil treated only.

The effect of taxifolin on the P-gp ATPase activity was evaluated by the Pgp-Glo ^TM^ assay. The results demonstrated that taxifolin significantly stimulated basal P-gp ATPase activity (Figure 2A). These results suggested that taxifolin acted as a P-gp ATPase stimulator and causing the inhibition of P-gp efflux function. The verapamil-stimulated P-gp ATPase activity was decreased by adding 0.1 μM to 20 μM of taxifolin (Figure 2B), suggesting taxifolin and verapamil were likely competed the same binding site on P-gp ATPase.

The inhibitory mechanism of interaction between taxifolin and human P-gp was further investigated in *ABCB1*/Flp-In^TM^-293 cell line. The P-gp fluorescent substrates rhodamine123 and doxorubicin were used in the efflux assays following Michaelis–Menten kinetics (Figure 2C,E). The efflux inhibition kinetics was further investigated by Lineweaver–Burk plot (Figure 2D,F). In rhodamine 123 efflux assays, both the maximum rate (Vmax) and the affinity (Km) were significantly reduced with increasing drug concentration of taxifolin (Table 1; Figure 2D). In terms of doxorubicin efflux assay, when the concentrations of taxifolin increased, the maximum rate (Vmax) and the affinity (Km) of doxorubicin efflux decreased (Table 1; Figure 2F). These results demonstrated that taxifolin uncompetitively inhibited the efflux of rhodamine 123 and doxorubicin by P-gp.

**Figure 3:**
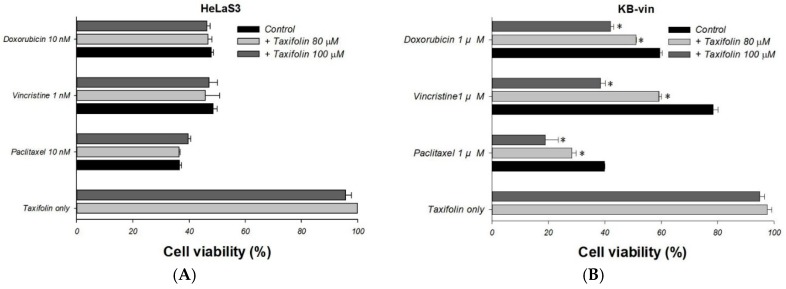

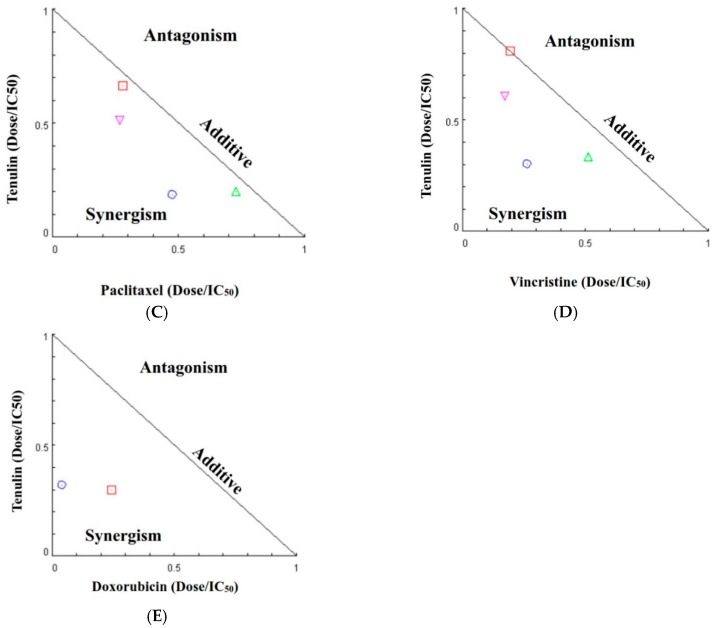
Resistance-reversal ability of taxifolin on MDR cancer cell line. (**A**,**B**) Cytotoxicity was conducted by SRB assay in HeLaS3 and KB-vin cell lines. Taxifolin significantly increased the cytotoxicity of paclitaxel, vincristine, and doxorubicin as compare to each chemotherapeutic agent alone. (**C**–**E**) The combination index represented the additive or synergistic effect of the combination treatment of taxifolin and chemotherapeutic agents. Data presented as mean ± SE of at least two experiments, * denotes *p* < 0.05 compared with alone treatment.

To study whether taxifolin could overcome the MDR to chemotherapeutic agents, we compared the cell viability of chemotherapeutic agents alone with the combination treatment in HeLaS3 and MDR KB-vin cell lines. Treatment of taxifolin alone showed no cytotoxic effect on either HeLaS3 or KB-vin, with more than 90% cell viability at 80 µM or 100 µM (Figure 3A,B). The cell viabilities of MDR cancer cell lines KB-vin were 60%, 80% and 40% after 72 h treatment of 1 µM doxorubicin, 1 µM vincristine and 1 µM paclitaxel, respectively. However, used in separate combinations with 80 μM and 100 μM taxifolin, the cell viabilities were significantly reduced (Figure 3B). Further investigated combination effects of taxifolin with chemotherapeutic agents, which were determined based on CI values calculated using CompuSyn software. The CI values and normalized isobologram of taxifolin (80 or 100 μM) with the three chemotherapeutic agents ranged from 0.36 to 1.00, suggesting either synergism or an additive effect of the combination treatments (Table 2; Figure 3C–E).

**Figure 4:**
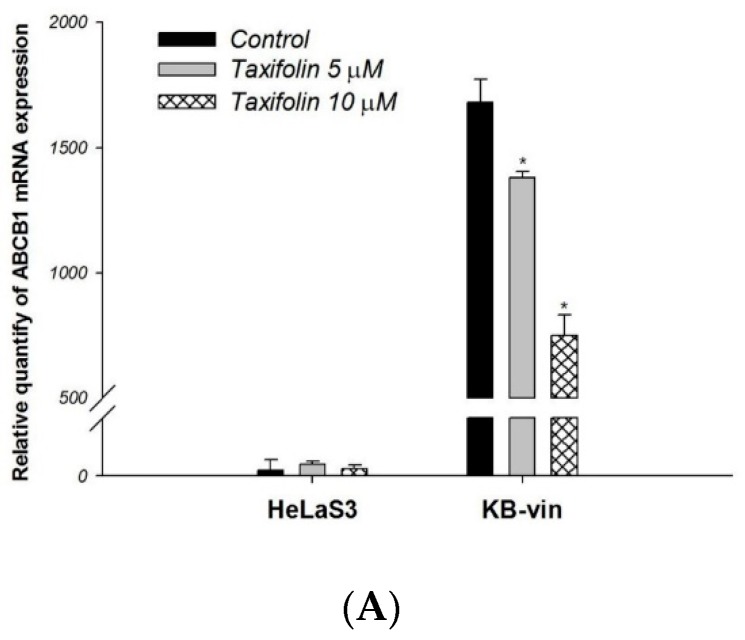

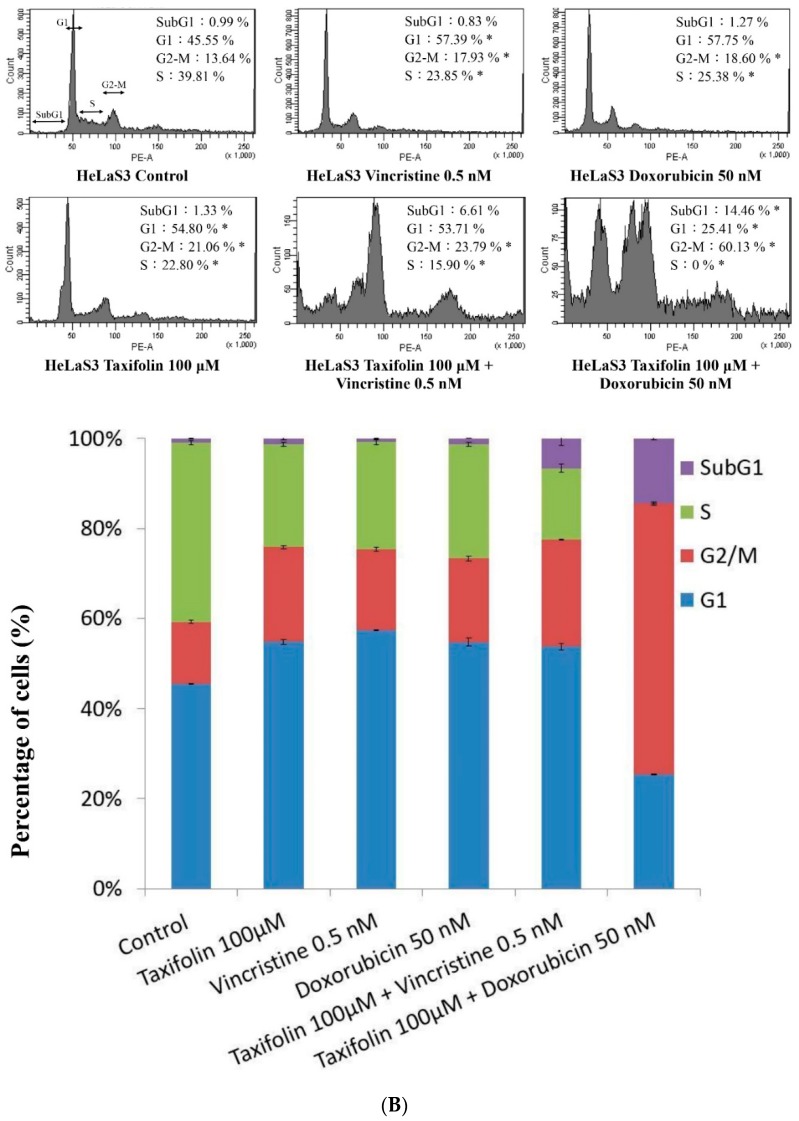

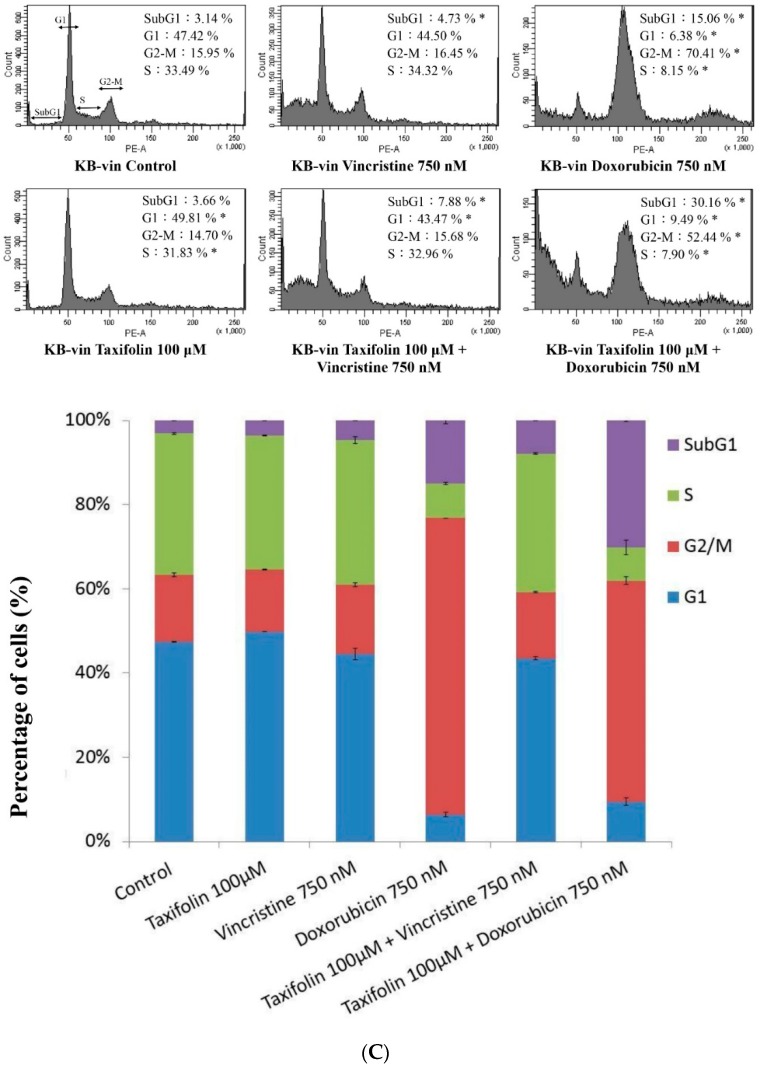
Effect of Taxifolin on P-gp mRNA expression and cell cycle analysis in HeLaS3 and KB-vin cells. Cells were treated with indicated concentration of compounds for 72 h. (**A**) *ABCB1* mRNA expression levels were significantly downregulated by taxifolin treatment in MDR KB-vin cancer cell line. (**B**,**C**) Combination of taxifolin with doxorubicin or vincristine increased levels of apoptosis (subG1) in HeLaS3 and KB-vin cells. Data presented as mean ± SE of at least two experiments, * denotes *p* < 0.05 compared with control group.

To further investigate the effects of taxifolin on *ABCB1* expression, we treated HeLaS3 cells and KB-vin cells with 5 μM or 10 μM taxifolin for 72 h and the expression were evaluated by real-time RT-PCR. Treatment of KB-vin with taxifolin for 72 h resulted in downregulation of *ABCB1* expression. This effect presented in a dose-dependent manner (Figure 4A). Cell cycle analysis demonstrated that taxifolin slightly increase doxorubicin-induced SubG1 arrest in HeLaS3 cells (Figure 4B). In KB-vin cells, taxifolin significantly increase either doxorubicin or vincristine inducing SubG1 arrest (Figure 4C).

### 2.3. The Docking Model of Taxifolin on P-gp

**Figure 5:**
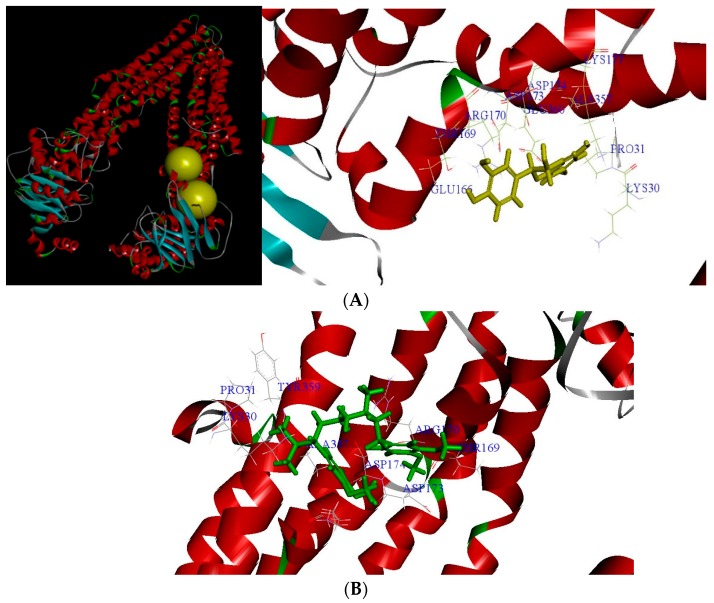

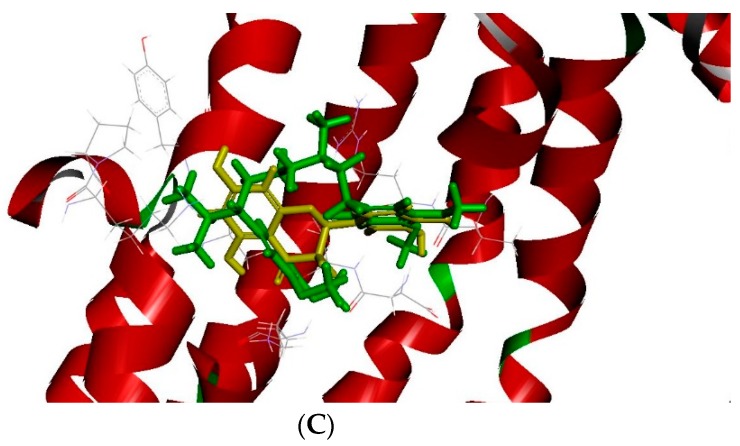
Molecular docking model P-glycoprotein antagonists. Superimposition of docked poses of compounds in the P-glycoprotein binding pocket of the X-ray structure (PDB id: 5KPI). (**A**) (**Left**) Bind pocket of P-gp; (**Right**) Docking pose of (−)-taxifolin in the active site of P-gp ligand binding domain. (**B**) Docking pose of verapamil in the active site of P-gp ligand binding domain. (**C**) Superposition of the docked pose of (−)-taxifolin (yellow atoms in dark green) and the X-ray pose of the agonist verapamil (green capped stick) in the active site of P-gp ligand binding domain. Hydrogen-bonding interactions are indicated by dashed ellipsoids.

We have performed docking study of (−)-taxifolin, quercetin, catechin and verapamil to the P-gp crystal structure (PDB id: 5KPI) [14]. The results demonstrated that the ligands with the best binding energies occupied the active sites of P-gp (Table 3). The binding energies of (−)-taxifolin, quercetin, and catechin are similar. As Figure 5A showed that 3D structure generated of P-gp bound to a ligand molecule (−)-taxifolin was used to study the ligand and receptor interactions. The binding mode clearly indicates the (−)-taxifolin of the ligand with the residues like ALA357, LYS 177, ASP174, GLU166, THR169, ARG170 and GLU360. In order to investigate whether taxifolin may compete with verapamil, we docked verapamil to the P-gp crystal structure using the same docking protocol. We found that (−)-taxifolin and verapamil fitted comfortably into the binding pocket for the agonist and the binding positions were similar (Figure 5B). Verapamil has similar form hydrogen bonds with any of these key residues of the activated form of ligand binding domain. The overall binding pose of verapamil was similar to the X-ray pose of (−)-taxifolin. The fluorine atom of the para trifluoromethyl group of verapamil formed a hydrogen bond with an amino group of the guanylyl moiety (LYS 177, THR169, ALA357, GLU166, ARG170, GLU360 and ASP174, as shown in Figure 5C). These computational results supported that taxifolin may compete with verapamil. By using computational approaches to preliminary screen hits from natural products and design novel small molecules may shorten drug discovery periods.

## 3. Discussion

P-gp overexpression remains one of the most probable mechanisms of multidrug-resistance, leading to the dilemma of cancer chemotherapy. The clinical application of the P-gp inhibitors was still limited due to unwanted toxicity or nonspecific effects. Natural products provide promising opportunities to resolve such problems. The flavonoids have been shown potential P-gp modulatory effects in several MDR cancer cell lines [8,13,15]. In the present study, taxifolin exhibited the most P-gp inhibitory effects in the primary screening. Among the mechanic study, taxifolin inhibited the P-gp efflux effect by stimulation of ATPase activity and interacted with P-gp transport of rhodamine 123 and doxorubicin via uncompetitive inhibition. Moreover, taxifolin re-sensitized the MDR cancer cell, KB-vin, to the chemotherapeutic agents by inhibition of *ABCB1* expression and enhancing the apoptosis. These results suggest that taxifolin is promising candidates to develop as a P-gp inhibitor for the synergistic cancer treatment.

Taxifolin is a dihydroflavonol which is abundant in fruits and plants. In addition to the antioxidant activity, taxifolin also exhibited anti-proliferative effect through increasing the mitotic arrest and apoptosis in human cancer cells [10,12]. Another study demonstrated that taxifolin inhibited chaperoning process of oncogenic proteins may play a potential role for cancer treatments [16]. As compared to other flavonoids, taxifolin exhibited comparable hepatoprotective activity to quercetin and superior activity to catechin [17]. Regarding the impact of taxifolin on ABC transporter, it has been reported that taxifolin affected MRP1-mediated transport activity [18]. However, limited studies have elucidated its ability on P-gp inhibition and MDR cancer reversing. Furthermore, the mechanisms and kinetics of the P-gp interaction is not clear. In present study, we demonstrated that taxifolin possessed P-gp inhibitory effects and exhibited the strongest MDR reversal ability in cancer cell under nontoxic concentrations. In the P-gp substrates identification, taxifolin did not enhance the fluorescence of UIC2 antibody. This result suggested that taxifolin was not a substrate of P-gp which was consistent with previous study [19]. The binding of taxifolin stimulated the P-gp ATPase activity, causing the inhibition of P-gp efflux function. These results suggested that taxifolin belongs to a Class II compound, which could enhance P-gp ATPase activity in a dose-dependent manner [20]. Taxifolin has been found to bind at ATP-binding site of heat shock protein 90 (Hsp90) [16]. The binding site of taxifolin on P-gp ATPase was further observed in the interaction with P-gp ATPase stimulator, verapamil. The verapamil-induced ATPase activity was inhibited by taxifolin, suggesting taxifolin may compete for the ATPase binding sites of verapamil. The kinetic mechanism was analyzed with two different P-gp substrates, rhodamine 123 and doxorubicin, to investigate the possible binding site of taxifolin on P-gp. There were two substrate-binding sites, the H-site and the R-site, and one modulator site, M-site, of P-gp. Both rhodamine123 and doxorubicin bind to the R-site, whereas rhodamine 123 has an additional binding pocket on the M-site [21]. The kinetic studies showed that taxifolin interacted with P-gp transport of rhodamine123 and doxorubicin via uncompetitive inhibition. Furthermore, the molecular docking results showed that taxifolin had higher affinity on P-gp active site. These results suggested that taxifolin may not directly compete with substrates but disturb the binding of P-gp and leading to P-gp efflux inhibition.

Flavonoids were potential agents for MDR cancers and it exhibited different reversal potencies toward different MDR cell lines [8,13,15,22]. The effect of taxifolin in reversing MDR cancers was evaluated by using vincristine-induced MDR cervical cancer cell lines, KB-vin. The results from cytotoxicity assay showed either synergism or an additive effect in combination treatment of taxifolin with chemotherapeutic drugs. This MDR reversal ability of taxifolin may contribute from the down regulation of *ABCB1* expression, as well as direct inhibited P-gp efflux function. Furthermore, taxifolin exhibited an additive apoptosis effect with vincristine and doxorubicin on a human MDR KB-vin cell line.

The strength of this study was the use of cells that stably overexpress human P-gp (*ABCB1*/Flp-In^TM^-293) to clarify the inhibitory effects and kinetic mechanisms of taxifolin. This transporter-specific system prevents the involvement of other transporters. In addition, we used a chemotherapeutic drug-induced MDR cancer cell line to evaluate the reversal ability and further confirm the tumor environment in clinical MDR circumstances. However, there were still some limitations in this study. Since previous evidences regarding the impacts of taxifolin on other types of ABC transporters [18,23,24,25], additional transporters could be included for the primary screening. The MDR reversal effect of taxifolin also needs to be further verified in animal study. The relatively high concentration of taxifolin was used as compared to other compounds or clinical agents. Since we attempted to identify a potent lead compound with P-gp modification effect from natural product, further structural modification was needed to develop the effective and safe agents.

## 4. Materials and Methods

### 4.1. Chemicals and Reagents

Dulbecco’s modified Eagle’s medium (DMEM), RPMI-1640 medium, 0.25% Trypsin-EDTA, phosphate-buffered saline (PBS) and fetal bovine serum (FBS) were all obtained from Thermo Fisher Scientific Inc. (Waltham, MA, USA). Calcein-AM, doxorubicin, vincristine, paclitaxel, rhodamine123, dimethyl sulfoxide (DMSO), R-(+)-verapamil, sulforhodamine B (SRB), trichloroacetic acid (TCA), Tris Base, taxifolin, luteolin, (−)-gallocatechin and (−)-catechin were purchased from Sigma Chemical Co (St. Louis, MO, USA).

### 4.2. Cell lines and Culture Condition

Human cervical carcinoma cell line HeLaS3 was purchased from Bioresource Collection and Research Center (Hsinchu, Taiwan). The KB-vin was multidrug resistant human cervical cancer cell line, was kindly gave from Dr. Kuo-Hsiung Lee (University of North Carolina, Chapel Hill, NC, USA). All cells were cultured in RPMI-1640 supplemented with 10% FBS at 37 °C in a humidified 5% CO_2_ incubator.

### 4.3. Cell Line Establishment

The establishment of human P-gp overexpression cells (*ABCB1*/Flp-In^TM^-293) was described in our previous study [26]. The cell culture maintenance and confirmation of P-gp expression were performed as previous study described [27].

### 4.4. Calcein-AM Accumulation Assay

The calcein-AM accumulation was perform to screen the effect of taxifolin on P-gp efflux activity as previous study with minor modifications [28]. Cells were pretreated with taxifolin, luteolin, (−)-gallocatechin or (−)-catechin for 30 min, and the fluorescence was measure by microplate reader.

### 4.5. Doxorubicin and Rhodamine123 Efflux Assay

These efflux assays were performed as previous study with minor modifications [29]. Cells were pretreated with or without 5 μM or 10 μM taxifolin for 30 min. After the efflux step, the supernatant were collected and the fluorescence was read immediately using microplate reader.

### 4.6. MDR1 Shift Assay

In order to evaluate whether taxifolin was the substrate of P-gp, the MDR1 shift assay was performed. UIC2 is a conformation-sensitive antibody against the extracellular conformational epitope of human P-gp while transporting substrates. The detailed steps for MDR1 shift assay were mentioned in previous study with minor modifications [30]. The cells were treated with taxifolin for 30 min and then performed the binding assays.

### 4.7. P-gp ATPase Activity Assays

The effects of taxifolin on P-gp ATPase activity was evaluated by the Pgp-GIO assay system (Promega, Madison, WI, USA) as described in previous study with minor modifications [30]. Taxifolin (from 0.1 to 20 μM) was tested with 25 μg of recombinant human P-gp membrane provided by the kit.

### 4.8. Cell Viability Assay and Drug Combination Assay

SRB assay were used to evaluate the reversal effects of taxifolin on human cancer cell lines as previously described with minor modifications [28]. Cells were treated with chemotherapeutic drugs (doxorubicin, vincristine and paclitaxel) with or without taxifolin for 72 h. The Chou–Talalay method and the CompuSyn software were used to determine the combination effects of taxifolin with chemotherapeutic drugs on cancer cell lines [31].

### 4.9. Cell-Cycle Analysis

Cell-cycle analysis was evaluated with PI/RNase Staining Buffer (Catalog No. 550825, BD Pharmingen™, Franklin Lakes, NJ, USA). Cells were treated with taxifolin at 37 °C for 72 h, and then washed with cold PBS followed by washing with Stain Buffer (Catalog No. 554656, BD Pharmingen™). Afterwards, cells were incubated with 0.5 mL of PI/RNase Staining Buffer and quantified the fluorescence by FACS analysis (BD FACSCanto II System).

### 4.10. RNA Extraction and Real-Time Quantitative RT-PCR

After 10 μM taxifolin treatment for 72 h, total RNA was isolated from the cells using the RNeasy Mini kit (Qiagen, Valencia, CA, USA). The cDNA were synthesized by the High-Capacity cDNA Reverse Transcription Kit (Life Technologies, Carlsbad, CA, USA). The real-time quantitative RT-PCR assays were applied to quantify *ABCB1* mRNA expression levels as described in previous study [27].

### 4.11. Docking Simulation

Docking was conducted by the CDOCKER docking method of Discovery Studio 4.5. The CDOCKER energy of best configuration docked into the interacting residues at active site region with P-glycoprotein from RCSB PDB (PDB id: 5KPI) and (−)-taxifolin were determined by the following Equation (1):

*E_binding_* = *E_compex_* − (*E_receptor_* + *E_ligand_*) (1)

### 4.12. Data and Statistical Analysis

For inhibitor kinetic analysis, the parameters were calculated by nonlinear regression by Scientist v2.01 (MicroMath Scientific Software, Salt Lake City, UT, USA) according to the Michaelis–Menten kinetic equation. Analysis of variance (ANOVA) with post hoc analysis were performed to detect the statistical differences. The p values less than 0.05 were considered as statistical significance.

## 5. Conclusions

Present study provide the information that taxifolin significantly inhibited human P-gp function through uncompetitive inhibition on doxorubicin and rhodamine123 and ATPase stimulation. Taxifolin also acted as a chemotherapeutic sensitizer in KB-vin MDR cancer cells. Further in vivo studies may provide additional evidence to support taxifolin used as a synergistic treatment for chemotherapy in clinical settings.

## Figures and Tables

**Table 1:** The effects of taxifolin on human P-gp-mediated efflux of rhodamine123 and doxorubicin in *ABCB1*/Flp-In^TM^-293 cells.

	Nonlinear Kinetic Parameters		
	**V_m_ (pmol/mg protein/10 min)**	**K_m_ (μM)**	
Nonlinear regression			
Rhodamine 123 only	12.22 ± 1.95	25.30 ± 4.99	
+taxifolin 5 μM	5.27 ± 0.24 *	10.93 ± 1.20 *	
+taxifolin 10 μM	3.20 ± 0.37 *	6.56 ± 1.53 *	
K_i_ from Lineweaver−Burk (μM)			3.65 ± 0.15
efflux IC50 (μM)			4.45 ± 0.22
	**V_m_ (pmol/mg protein/120 min)**	**K_m_ (μM)**	
Nonlinear regression			
Doxorubicin only	48.38 ± 4.98	37.56 ± 4.89	
+taxifolin 5 μM	23.28 ± 2.63 *	18.06 ± 3.23 *	
+taxifolin 10 μM	16.57 ± 3.71 *	13.72 ± 4.73 *	
K_i_ from Lineweaver−Burk (μM)			5.19 ± 0.56
efflux IC50 (μM)			4.50 ± 0.31

V_m_, the maximal efflux rate; K_m_, the Michaelis–Menten constant. * *p* < 0.05 as compared with rhodamine123 or doxorubicin only.

**Table 2:** Combination index analysis of vincristine, doxorubicin and paclitaxel combined with teaxifolin at a non-constant ratio in MDR KB-vin cells.

Chemotherapeutic Agent (nM)	Taxifolin (μM)	Fa ^a^	CI ^b^	Pharmacological Effect
**Paclitaxel**				
1000	80	0.27	0.93	Additive
	100	0.14	0.66	Synergism
100	80	0.81	0.78	Moderate synergism
	100	0.82	0.95	Additive
**Vincristine**				
1000	80	0.58	0.85	Moderate synergism
	100	0.37	0.56	Synergism
100	80	0.87	0.78	Moderate synergism
	100	0.89	1.00	Additive
**Doxorubicin**				
1000	80	0.51	0.55	Synergism
	100	0.41	0.36	Synergism

^a^ Fa: Fraction affected; ^b^ CI: Combination Index.

**Table 3:** The -CDOCKER Energy score of screened compounds.

PubChem CID	Chemical Names	-CDOCKER Energy (kcal/mole)
712316	(–)-Taxifolin	32.12
2520	Verapamil	15.7402
5280343	Quercetin	28.62
9064	Catechin	28.16

## Abbreviations

MDR	multidrug resistance
ABC	ATP-binding cassette
P-gp	P-glycoprotein
